# Proteome Analysis of PC12 Cells Reveals Alterations in Translation Regulation and Actin Signaling Induced by Clozapine

**DOI:** 10.1007/s11064-021-03348-4

**Published:** 2021-05-23

**Authors:** Urszula Jankowska, Bozena Skupien-Rabian, Bianka Swiderska, Gabriela Prus, Marta Dziedzicka-Wasylewska, Sylwia Kedracka-Krok

**Affiliations:** 1 Proteomics and Mass Spectrometry Core Facility, Malopolska Centre of Biotechnology, Jagiellonian University, Gronostajowa 7a str, 30-387 Krakow, Poland; 2 Mass Spectrometry Laboratory, Institute of Biochemistry and Biophysics Polish Academy of Sciences, Pawinskiego 5a, Warsaw, Poland; 3 Department of Physical Biochemistry, Faculty of Biochemistry, Biophysics and Biotechnology, Jagiellonian University, Gronostajowa 7, Krakow, Poland

**Keywords:** Actin signaling, Clozapine, mTOR, Antipsychotic drugs, Proteome, Translation

## Abstract

Although antipsychotics are routinely used in the treatment of schizophrenia for the last decades, their precise mechanism of action is still unclear. In this study, we investigated changes in the PC12 cells’ proteome under the influence of clozapine, risperidone, and haloperidol to identify protein pathways regulated by antipsychotics. Analysis of the protein profiles in two time points: after 12 and 24 h of incubation with drugs revealed significant alterations in 510 proteins. Further canonical pathway analysis revealed an inhibition of ciliary trophic factor signaling after treatment with haloperidol and showed a decrease in acute phase response signaling in the risperidone group. Interestingly, all tested drugs have caused changes in PC12 proteome which correspond to inhibition of cytokines: tumor necrosis factor (TNF) and transforming growth factor beta 1 (TGF-β1). We also found that the 12-h incubation with clozapine caused up-regulation of protein kinase A signaling and translation machinery. After 24 h of treatment with clozapine, the inhibition of the actin cytoskeleton signaling and Rho proteins signaling was revealed. The obtained results suggest that the mammalian target of rapamycin complex 1 (mTORC1) and 2 (mTORC2) play a central role in the signal transduction of clozapine.

**Supplementary Information:**

The online version contains supplementary material available at 10.1007/s11064-021-03348-4.

## Introduction

Antipsychotics are commonly used for treating schizophrenia, bipolar disorder, and other psychotic diseases [2]. Treatment of schizophrenia is considered one of the greatest challenges of modern clinical psychiatry, as around 30% of patients do not respond to pharmacotherapy and it is associated with the risk of serious side effects [3].

Although the receptor profile of antipsychotics is relatively well understood, the precise mechanism of these agent’s action remains unclear. Different therapeutic effects of drugs are probably related to their diverse affinity for individual receptors, but another explanation is based on the observation that the conformational changes of the receptor may be different depending on the ligand, leading to the activation of different signal transduction cascades [4]. All antipsychotics block the dopamine D2 receptor and increase the pathway of cyclic AMP/protein kinase A (PKA) [2]. The other pathways relevant to antipsychotics pharmacology are: the protein kinase B (Akt)/glycogen synthase kinase pathway, the mitogen-activated protein kinase cascade, and the β-arrestin-2-dependent signaling [5, 6]. The pathways interplay at various levels of signal transduction, i.e., secondary messengers, signaling proteins/kinases/phosphatases, and transcription factors, which generates a complex system of mutual dependencies [5].

Clozapine has a multireceptor binding profile, including affinity for serotonergic, dopaminergic, and muscarinic receptors [7]. It is the first atypical drug, introduced to clinics in the 1970s, and remains the most effective antipsychotic drug to the present day [8]. Clozapine demonstrates effectiveness not only for treating the positive, but also to a certain degree negative and cognitive symptoms (improved verbal fluency) of schizophrenia with a greatly reduced tendency to induce extrapyramidal side effects and tardive dyskinesia [9]. However, due to the risk of serious side effects such as potentially life-threatening agranulocytosis, metabolic disorders, and myocarditis, clozapine is prescribed rarely, mainly in treatment-resistant schizophrenia. Its introduction to the therapy was the last crucial innovation in psychopharmacology of schizophrenia [10]. The lack of breakthrough since then indicates that novel therapeutics should be aimed at cellular and molecular targets, rather than just the dopamine or serotonin receptors [8].

Therefore, this study aimed to identify protein pathways regulated by antipsychotics with different mode of action (haloperidol, risperidone, and clozapine) using quantitative proteomics methods. Haloperidol is a representative of older, typical antipsychotics and exhibits high affinity dopamine D2 receptor antagonism. Risperidone is considered atypical, nonetheless, it has intermediate properties between both groups of antipsychotics.

In this study, the effect of antipsychotics was examined on cells derived from a pheochromocytoma of rat adrenal medulla (PC12 cells). The PC12 cell line is one of the most valuable mammalian cell model commonly used to study nervous system disorders and the mechanisms of drug action [11, 12]. The PC12 cells respond to the nerve growth factor and are able to synthetize and store neurotransmitters [13]. The choice of PC12 cells was also dictated by the presence of antipsychotic receptors: D1, D2, D4, 5HT2A, 5HT3, M1, M4, M5 [14–18].

Therefore, in the presented study, we investigated the alterations in the protein profile of PC12 cells induced by clozapine, risperidone, and haloperidol to provide insights into further stages of signal transmission for antipsychotics. Distinguishing the crucial pathways underlying antipsychotics’ activity may contribute to developing novel medications with greater efficacy and improved tolerability.

Quantitative proteomics was carried out using two approaches: iTRAQ-based and 2D-DIGE. The methods complement each other, allowing to get a broader picture of PC12 cells’ proteome. To observe the changes at various stages of signal transduction, the proteomic analysis was performed at two time points (12 and 24 h of incubation). The Ingenuity Pathway Analysis (IPA) was used to identify protein interaction networks and signaling pathways activated or inhibited under the influence of drugs.

## Materials and Methods

### Cell Culture and Protein Sample Preparation

PC12 cells (American Type Culture Collection) were grown in F12K medium (Sigma-Aldrich) supplemented with 15% horse serum and 2.5% FBS (Sigma-Aldrich). Upon reaching ~ 80% confluency, the media were changed to media containing one of the drugs: clozapine (10 μM), haloperidol (3 μM), risperidone (3 μM) or vehicle solution (0.33% ethanol, Sigma-Aldrich). The concentrations of haloperidol and risperidone were according to works of Kashem et al. and Ahmed et al. [19, 20]. The clozapine concentration was based on works of Tejedor-Real et al. and Weintraub et al. [21, 22]. Tejedor-Real et al. showed that the reduced level of tyrosine hydroxylase observed in the rat brain after clozapine administration was obtained in PC12 cells already at the drug concentration of 10 μM [21]. After 12 or 24 h of incubation, the cells were washed twice with 5 mM magnesium acetate in 10 mM Tris, pH 8.0 and lysed directly on a culture dish in ice-cold lysis buffer (7 M urea, 2 M thiourea, 4% CHAPS, 30 mM Tris pH 8.5). The extracts were sonicated at 320 W, 20 kHz, 30 s/30 s on/off for 15 min in a Bioruptor UCD-200 (Diagenode), centrifuged at 20,000*g* for 15 min at 15 °C and supernatants were collected. The experiment was performed in six replicates, between passages 6 and 12. The protein concentration was determined using the Bradford assay [23]. To facilitate normalization and quantification of the data, an internal standard (IS) was prepared by combining 75 μg of protein mixture from each sample (48 samples).

### iTRAQ

#### Protein Digestion, iTRAQ Labeling, and Peptide Fractionation by IEF

For the 8-plex iTRAQ labeling (Applied Biosystems), 25 μg of protein mixtures from two biological replicates were pooled. Protein digestion and peptide labeling was performed on spin columns with a 30-kDa membrane cutoff (Vivacon500, Sartorius Stedim) according to the iFASP procedure (isobaric mass tagging with filter-aided sample preparation) [24] with some modifications. A labeling scheme is shown in Supplementary file 1. Salts and excess reagents were removed from the combined filtrates by solid phase extraction on C18 Extraction Disk Cartridges (Empore 7 mm dia./3 ml vol., Sigma). To minimize the precursor co-isolation occurrence, peptides were separated by IEF into 34 fractions on 24 cm linear pH 3–10 Immobiline DryStrips (GE Healthcare). Peptides were extracted from the gels, vacuum-dried, and purified on C18 StageTips [25]. A detailed procedure is presented in Supplementary file 1.

#### LC–MS/MS of Labeled Peptides

Each fraction was analyzed in two technical replicates by LC–MS/MS with an UltiMate 3000RSLCnano System (Thermo Scientific) coupled via a Digital PicoView 550 nanospray source (New Objective) to a Q-Exactive (Thermo Scientific) mass spectrometer. Peptides were injected into a precolumn (AcclaimPepMap100 C18, 2 cm × 75 μm, 3 μm, 100 Å) using 4% ACN with 0.05% TFA as the mobile phase and further separated on an analytical column (AcclaimPepMapRLSC C18, 50 cm × 75 μm, 2 μm, 100 Å) with a 4–44% ACN gradient in the presence of 0.05% formic acid for 120 min at a flow rate of 300 nl/min. The Q-Exactive was operated in a data-dependent mode using a top ten method at 33% of normalized collision energy (NCE) and 30 s of dynamic exclusion. Full scan MS spectra were acquired from 350 to 1800 m/z with a resolution of 70,000 at m/z 200 and using an automatic gain control (AGC) target of 1e6. The MS/MS spectra were acquired with a resolution of 17,500 at m/z 200 with an AGC target of 5e4. The maximum ion accumulation time for the full MS and MS/MS scans was 100 ms. The lock mass option was enabled for survey scans to improve mass accuracy. The MS proteomics data have been deposited to the ProteomeXchange Consortium via the PRIDE [1] partner repository with the dataset identifier PXD014422.

#### Data Analysis

The Proteome Discoverer platform (v. 1.4, Thermo Scientific) was used for identification and preliminary quantification of proteins. The data obtained from both technical replicates were searched together against the Swiss Prot_201506 database with taxonomic restriction to Rodentia (26 322 sequences) using an in-house Mascot server (v. 2.5.0, Matrix Science) with the following parameters: digestion by trypsin with a maximum of two missed cleavages; iTRAQ 8-plex (K, N-terminus) and carbamidomethylation (C) as fixed modification; oxidation (M) and deamidation (NQ) as variable modifications. Mass tolerance for the precursor and fragment ions was set to 10 ppm and 20 mmu, respectively. The FDR for peptides was calculated by a target–decoy approach and was set to 1%. Percolator algorithm was used to assess the reliability of protein identification. During the quantitative analysis, a "quan value correction" was used, related to the incomplete purity of the individual markers. Quantitative information was only obtained from unique peptides with the co-isolation below 50%. A quantitative analysis combining all results was carried out in the Scaffold Q + (version 4.4.7, Proteome Software Inc.). Threshold of 95% probability was used for peptide and protein identification. Protein probabilities were assigned by the Protein Prophet algorithm [26]. In addition, protein identification required at least two identified peptides. Proteins that shared the assigned peptides with other entries and could not be differentiated were grouped to satisfy the principles of parsimony. Channels were corrected in all samples according to the algorithm described in i-Tracker [27]. Acquired intensities in the experiment were globally normalized across all acquisition runs. Individual quantitative samples were normalized within each acquisition run. Intensities for each peptide identification were normalized within the assigned protein. The reference channels from IS (113) were normalized to produce a 1: onefold change. All normalization calculations were performed using averages to multiplicatively normalize data.

### DIGE

#### Cyanine Dye Labeling and Protein Separation by 2DE

Six replicates from each group were analyzed by DIGE. Lysates containing 800 µg of protein were purified by methanol/chloroform precipitation according to the previous protocol [28]. Pellets were dissolved in the lysis buffer. Protein concentrations were measured by the Bradford assay and adjusted to 4.5 mg/ml. Labeling of the proteins and separation by 2DE was executed as described in our earlier work [29]. Briefly, 33 μg of protein from each sample was labeled with 110 pmol of Cy3 or Cy5 and the IS was labeled with Cy2. Samples were combined according to Table S1 in Supplementary file 1 and loaded onto 24 cm nonlinear pH 3–10 Immobiline DryStrips (GE Healthcare). IEF was performed on a PROTEAN® i12™ IEF System for a total of 78 000 Vhr. SDS-PAGE was carried out using an Ettan DALTsix Large Vertical System (GE Healthcare). Fluorescent images of the DIGE gels were obtained using the Typhoon Trio + Imager (GE Healthcare) at a resolution of 100 dpc. Next, the gels were stained with a lab-made ruthenium II tris-(bathophenanthroline disulfonate) and scanned again [30].

#### Gel Image Analysis

The evaluation of the DIGE gel patterns was performed using the difference in gel analysis mode and the biological variation analysis mode of DeCyder software v7.2 (GE Healthcare) as described previously [29]. Detection algorithm 6.0 was used with an estimated number of spots set to 10 000. In order to exclude artifacts, spots which volume value was less than 30 000 were rejected. Correct matching of the spots across the gels was manually checked.

#### Protein Identification from Gel Spots by LC–MS/MS

The selected spots were cut using an automatic Ettan SpotPicker (GE Healthcare) and in gel digested with trypsin as reported previously [31]. Protein identification was performed on the LC–MS system featured above (iTRAQ section) with the following modifications. Peptides were loaded onto the precolumn using 2% ACN with 0.05% TFA and separated with a 30-min gradient 2–40% ACN in 0.05% formic acid on a 15 cm analytical column (AcclaimPepMapRLSC, 15 cm × 75 μm, C18, 2 μm, 100 Å). The top six method was used with NCE set to 27. Full scan MS spectra were acquired with a resolution of 70,000 at m/z 200 with an AGC target of 1e6. The MS/MS spectra were acquired with a resolution of 35,000 at m/z 200 with an AGC target of 5e5. The maximum ion accumulation time for the full MS and MS/MS scans was 120 ms. The RAW MS files were processed by the Proteome Discoverer platform and searched against the Swiss Prot_201505 database restricted to Rodentia taxonomy (26,248 sequences) with the following parameters: digestion by trypsin with maximum one missed cleavage; carbamidomethylation (C) as fixed modification; oxidation (M) and phosphorylation (STY) as variable modifications; peptide mass tolerance ± 10 ppm and fragment mass tolerance ± 20 mmu. Only proteins identified with at least two peptides and with a Mascot score value over 90 were accepted. Each identification was confirmed from at least two gels. Only unambiguous identifications were accepted.

### Ingenuity Pathway Analysis (IPA)

The differential proteins identified with iTRAQ and DIGE methods were combined and imported into the IPA platform (Ingenuity® Systems; http://www.ingenuity.com). The computational algorithms of the software use annotation of the biological literature to identify biological functions, protein interaction networks, and signaling pathways regulated in the same way (activated or inhibited). The analysis of upstream regulators includes determining proteins (or small molecule compounds, miRNAs), which can be responsible for observed changes in the proteome. The great advantage of the program is considering not only the type of the protein, but also the direction of the observed alterations.

### Statistical Analysis

Statistical significance of the differentially expressed proteins was assessed using the Student’s t-test. Calculations were performed with Scaffold Q + and with Biological Variation Analysis module of Decyder 7.0 for iTRAQ and DIGE experiments, respectively. Proteins whose relative expression was changed > 1.17-fold across groups at the 95% confidence level were considered significant (*p* value  < 0.05 and FC > 1.17 or FC < − 1.17). This assumed that even slight variations in the levels of multiple proteins can result in pathway alterations. Overlap of observed and predicted regulated protein sets in the IPA was calculated using the Fisher's exact test (significance threshold: *p* value < 0.05). Activation z-score was computed to predict regulation patterns as inhibition (z-score ≤ − 2) or activation (z-score ≥ 2) [32].

## Results and Discussion

**Fig. 1:**
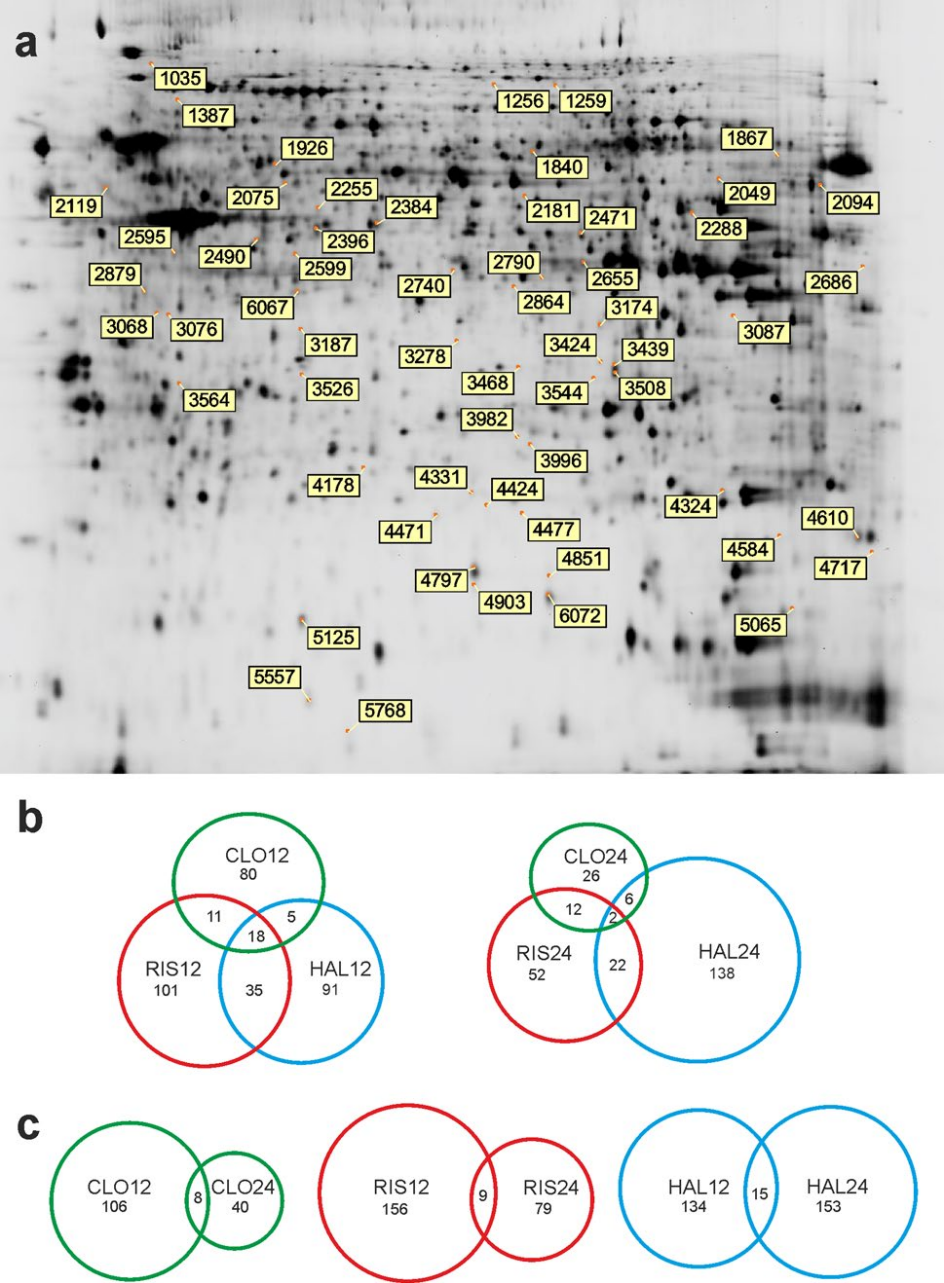
Representative DIGE gel image of internal standard from PC12 cells with marked differential proteins after treatment with studied antipsychotic drugs (**A**). Venn diagrams demonstrating the overlap between differential proteins found after treatment with clozapine (CLO), risperidone (RIS), haloperidol (HAL) (**B**), regarding the time of incubation (12 h/24 h) (**C**)

In this study, we aimed to explore alterations in the protein profile of PC12 cells after incubation with antipsychotic drugs: clozapine, risperidone, and haloperidol for 12 and 24 h. Six biological replicates of the control and treated samples were analyzed with two high-throughput proteomics approaches: iTRAQ and DIGE. Proteins from cell lysates were digested, labelled with 8-plex iTRAQ tags, and analyzed by the Orbitrap Q-Exactive mass spectrometer. As a result, on average 4 523 ± 866 proteins were identified. Quantitative analysis was performed on 3117 proteins with at least two unique peptides assigned with the use of Scaffold Q + software. Signal intensities of peptides in individual samples were normalized (Supplementary file 1, Fig. S1). There were 510 differential proteins found (Supplementary file 2). With the DIGE method 48 samples (and IS) were separated on 24 gels based on their size and pI values. On each gel, on average, 5240 ± 550 spots were detected and about 70% of them were matched and quantified. For each spot, the ratio of the normalized mean spot volume relative to the control was calculated. We found significant alterations in 78 spots. Proteins were unambiguously identified in 59 spots (Supplementary file 2), the position of which is shown on a representative electrophoretic gel image (Fig. 1a). The differential proteins identified by the iTRAQ method were distinct from those obtained by the DIGE method. Only two proteins, cofilin-1 and destrin, were found to be differentially expressed by both methods. In the case of cofilin-1, the upregulation after clozapine treatment was detected using the iTRAQ approach, while DIGE showed downregulation of this protein in response to risperidone and haloperidol. For destrin, the iTRAQ analysis showed the higher abundance of this protein after incubation with clozapine and risperidone. On the other hand, the lower level of destrin was revealed for haloperidol as well as risperidone using DIGE. The discrepancy in the direction of change for risperidone may be concerning, however, results from the gel-based approach may not refer to the outcome of the iTRAQ analysis as two methods quantify different pools of proteins. The peptide-level quantification used in iTRAQ provides information about the protein abundance, but without distinction between the forms of protein, while in DIGE protocol protein species (i.e. isoforms, modified forms) are separated and quantified before the enzyme digestion [33]. Thus, a given protein can be present in several spots, which was also the case in our experiment (see examples in a table in Supplementary file 2). The complementary nature of DIGE and iTRAQ methods has also been shown in other proteomics studies [34–36].

### Shared Changes in PC12 Proteome Induced by Clozapine, Risperidone, and Haloperidol

Venn diagram analysis indicated that very few proteins demonstrated overlapping changes induced by all studied antipsychotics (Fig. 1b, c). Only 23 proteins with altered expression were common to clozapine, risperidone, and haloperidol (excluding the incubation time factor). Shared differential proteins with a brief description of their functions were presented in Table 1. Interestingly, most of shared alterations were identified after 12 h of drug incubation, suggesting that drugs generally share only initial step in signal transduction pathways. A common observation among groups was downregulation of plasminogen activator inhibitor 1 (SERPINE1), which is a part of the senescence-associated secretory phenotype (SASP) and is involved in controlling inflammation, which is significant risk factor of schizophrenia [37, 38]. Enhanced SERPINE1 levels are associated with many pathophysiological processes including metabolic disturbances, insulin resistance syndrome, chronic stress and development of cardiovascular disease [39]. Next common alteration after 12 h of incubation with drugs was downregulation of matrix metalloproteinase-14 (MMP14), which together with SERPINE1 controls extracellular matrix proteolysis [40]. Metalloproteinases play an important role in schizophrenia and other neuropsychiatric disorders probable by abnormal synaptic plasticity and functional reorganization of excitatory synapses that are located on dendritic spines [41]. Moreover, metalloproteinases influence learning and memory processes and are linked to progression of neuroinflammatory disorders [42].

**Table 1:**
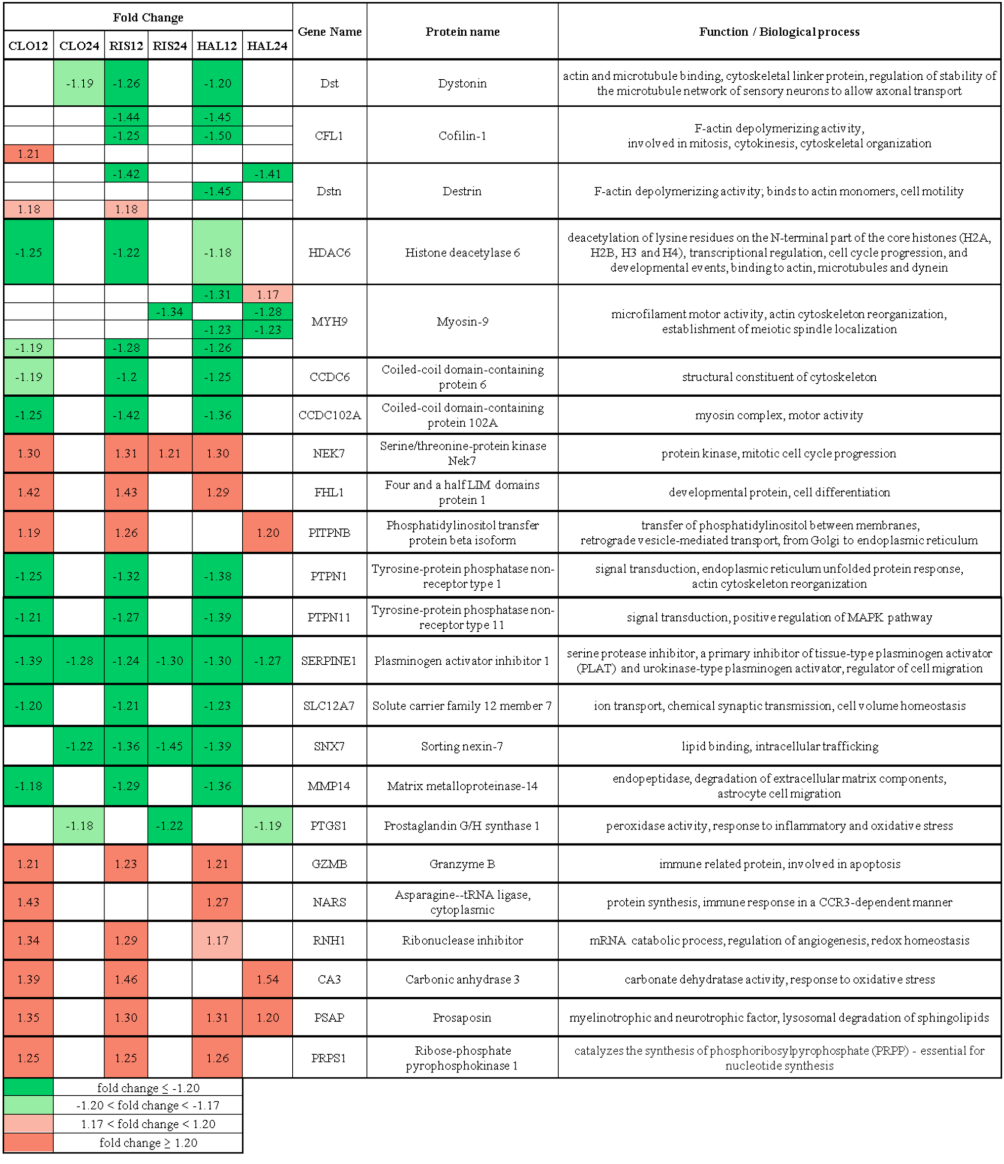
Fold change of differential proteins detected in PC12 cells shared between drugs: clozapine (CLO), risperidone (RIS), and haloperidol (HAL) revealed by iTRAQ or DIGE methods

Proteins MYH9, CFL1, Dstn have been identified in several spots on the gels

**Fig. 2:**
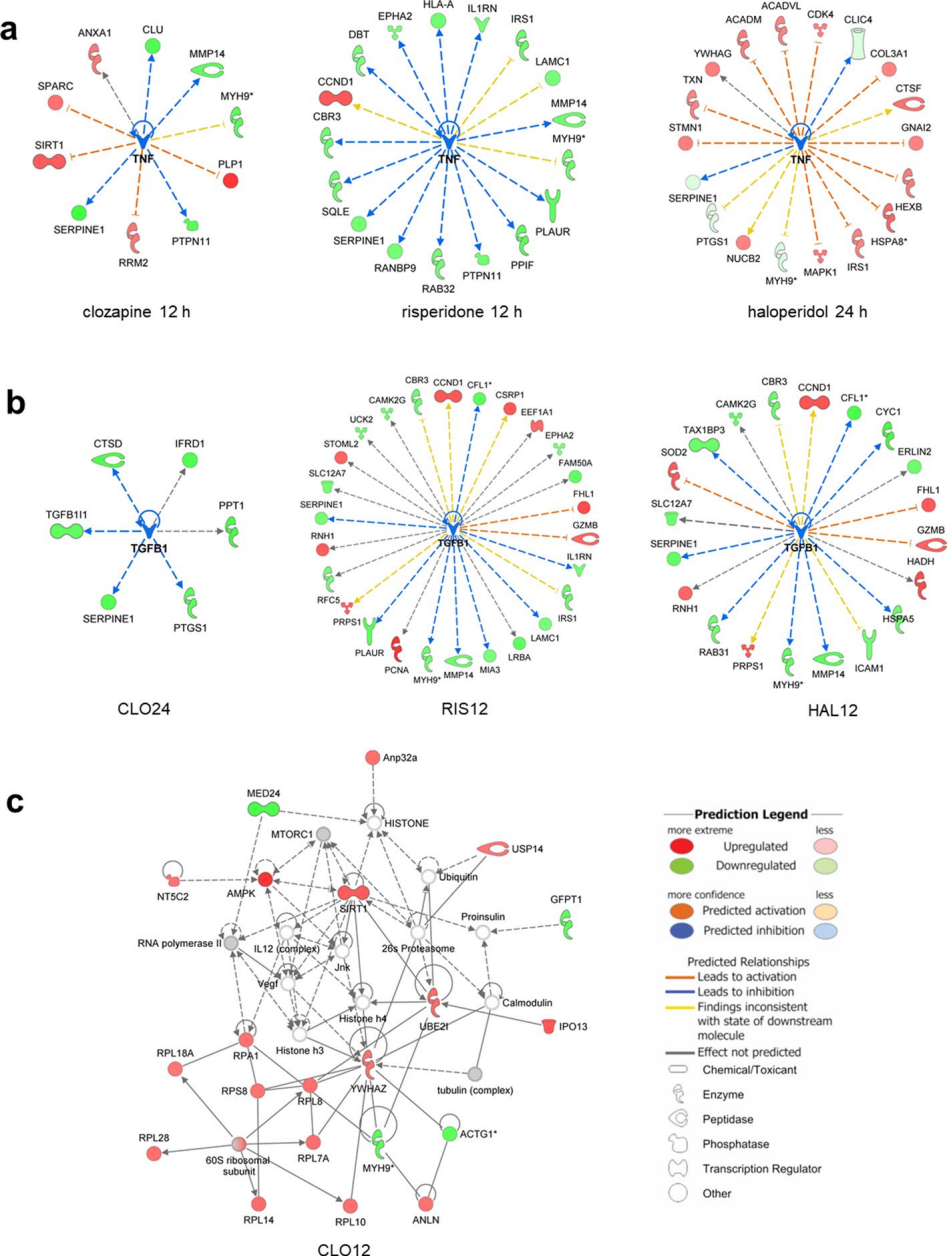
Tumor necrosis factor (TNF) as the primary regulator of cell changes after incubation with antipsychotic drugs: clozapine 12 h (CLO12), z-score = − 2.42; risperidone 12 h (RIS12), z-score = − 2.04; haloperidol 24 h (HAL24), z-score = − 2.45 (**A**). Transforming growth factor beta 1 (TGF-β1) as an upstream regulator of proteome changes after incubation with antipsychotics: clozapine 24 h (CLO24), z-score = − 1.96; risperidone 12 h, z-score = − 1.88; haloperidol 12 h (HAL12), z-score = − 2.16 (**B**). Emerged network identified by IPA based on differential proteins after 12 h of incubation with clozapine (**C**). Full and dashed lines depict direct and indirect connections, respectively

For a broader interpretation of the results, a functional analysis on several levels was performed in IPA: pathway enrichment, upstream regulators, functional networks. Detailed results are presented in Supplementary file 3. The canonical pathway analysis in IPA revealed eight signaling pathways with statistically significant alteration in at least one study group, which are presented in Table 2. The upstream regulator analysis indicated that changes in PC12 proteome under the influence of all tested antipsychotics correspond to molecular changes typical for inhibition of cytokines: TNF and TGF-β1 (Fig. 2ab). Interestingly, this conclusion was made on the basis of mostly different proteins in each group, what suggests that antipsychotics affects proteins engaged in inflammatory process, but in a different way. Increased TNF-α and other inflammatory markers, have been associated with negative symptoms of schizophrenia [43]. Despite many studies that confirm a decrease in cytokine levels in response to antipsychotic drugs [44], there are also contradictory reports [45]. Moreover, the immunomodulatory role of antipsychotics is much more complicated than just restoration of retained cytokines to normal levels [46].

**Table 2:**
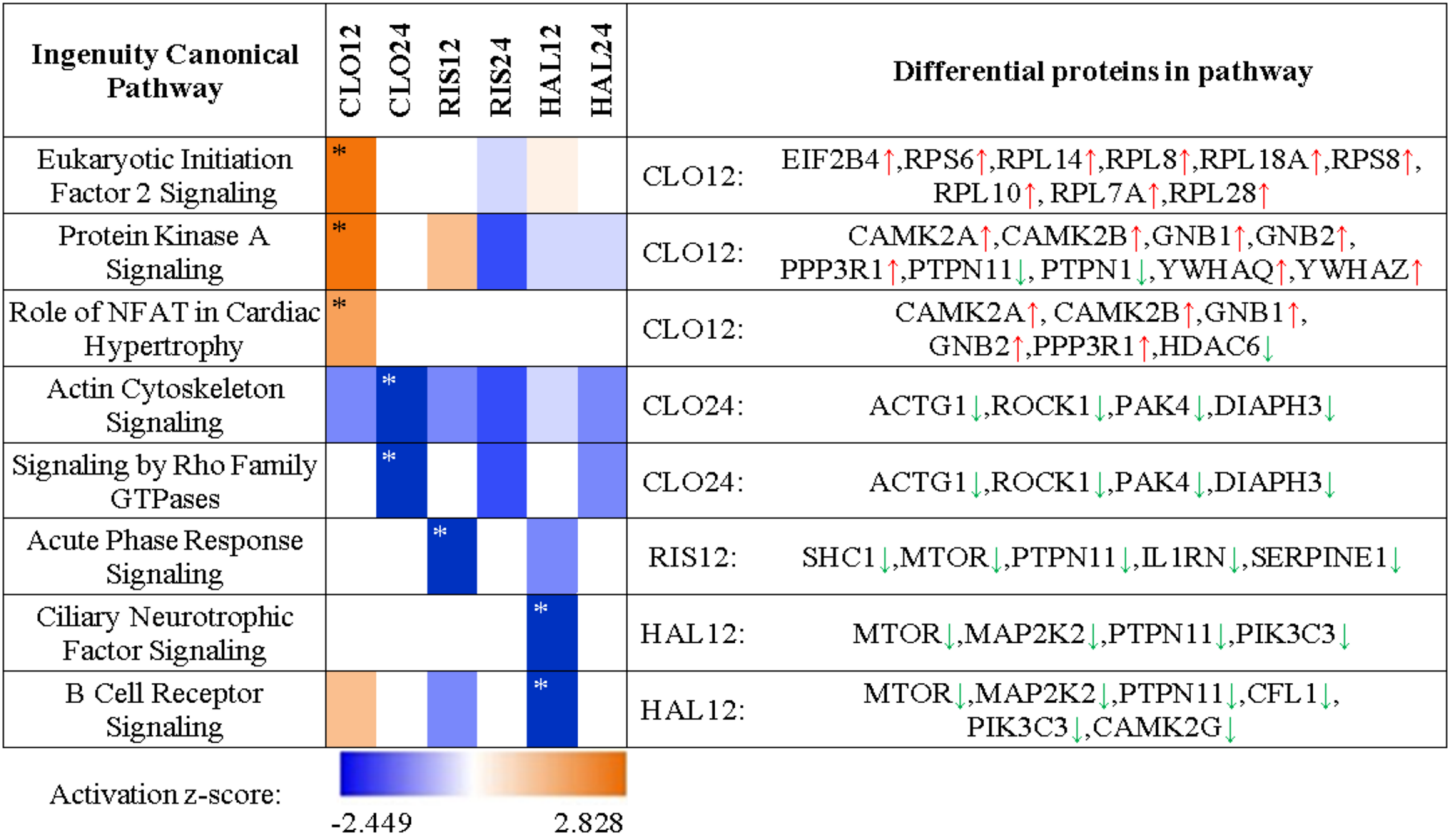
Signal transmission pathways inhibited (blue) or activated (orange) under the influence of 12 or 24 h treatment with clozapine (CLO), risperidone (RIS), and haloperidol (HAL)

Table generated through the use of IPA

*Significant regulation: z-score ≤ − 2.00 or ≥ 2.00 and p-value < 0.01

### Haloperidol Induced the Inhibition of the Ciliary Trophic Factor Signaling

Canonical pathways analysis revealed inhibition of the ciliary neurotrophic factor (CNTF) signaling after 12-h incubation with haloperidol (z-score = − 2.00, Table 2), what was based on downregulation of the following proteins: mTOR kinase (MTOR), MAP kinase kinase 2 (MAP2K2), tyrosine-protein phosphatase non-receptor type 11(PTPN11), PI3-kinase type 3 (PIK3C3). CNTF belongs to the interleukin-6 (IL-6) cytokine family which promotes neurite outgrowth in the hypothalamus and hippocampus [47], increases neuronal survival after injury [48] and enhances cognitive and memory function in rodent models [49, 50]. CNTF also regulates D2-receptor-dependent adult neurogenesis, so modulating CNTF has been suggested as potential therapeutic strategy for normalizing dopaminergic and neurogenic deficits [51]. The association of CNTF-encoding gene polymorphism with schizophrenia has been debated since the 1990s [52–54]. Certain types of CNTF-encoding gene polymorphisms cause patients to respond positively to treatment with iloperidone, an atypical antipsychotic [55]. It has been postulated by Mori et al. that D2 receptor activation stimulates CNTF expression [51], thus it could be expected that haloperidol, D2 receptor antagonist, may have a negative impact on CNTF level.

### Risperidone had an Inhibitory Effect on the Acute Phase Response Signaling

The canonical pathway analysis of proteome changes induced by risperidone, indicated the inhibition of the acute phase response signaling (z-score = − 2.24, Table 2). The conclusion was drawn from the observed downregulation of proteins produced in response to inflammation: interleukin-1 receptor antagonist protein (IL1RN), SHC-transforming protein 1 (SHC1), mTOR kinase (MTOR), plasminogen activator inhibitor 1 (SERPINE1). Their concentration has also been shown to be higher in schizophrenia patients [56–58]. Cytokine IL1RN modulates a variety of interleukin 1 related immune and inflammatory responses. It is released in response to IL-1β and IL-6 to reduce the action of IL-1 by binding to its receptor [59]. The elevation of IL1RN protein has been shown in schizophrenic patients [60–62]. However, there are also reports of a reduction in IL1RN levels in the prefrontal cortex of schizophrenic patients [63]. It was shown that the risperidone therapy caused a decrease in the level of IL1RN [64], while an increase in the level of IL1RA was found after treatment with haloperidol and clozapine [65, 66]. In another study, the level of the protein did not change under the influence of various antipsychotics [67]. The inhibitory effect of risperidone on proinflammatory signaling is also supported by another type of IPA analysis. Searching for upstream regulators revealed that the number of changes detected in our experiment can be explained by downregulation of TNF and TGF-β1 cytokines (Fig. 2a, b).

### Alterations in PC12 Proteome Induced by Clozapine

The common mechanism of antipsychotic drug action is dopamine D2 receptor blockage and resulting activation of cyclic AMP/protein kinase A pathway. Numerous reports showed the increase of PKA signaling induced by clozapine and other antipsychotics [68–70]. It was also observed in studies on rats that haloperidol and clozapine impact PKA phosphorylation differently and depending on the method of drug administration—single or chronic [68]. Here, the canonical pathway analysis showed that after 12 h of incubation with clozapine (CLO12 experimental group), the protein kinase A signaling was still activated (PKA, z-score = 2.83, Table 2). PKA controls a variety of processes through phosphorylation of transcription factor CREB, protein phosphatases, glutamatergic receptors, and it is involved in mammalian target of rapamycin (mTOR) activation [71, 72]. PKA signaling has been also implicated in the pathology of schizophrenia [73].

Part of the differential proteins assigned to the PKA signaling was also related by the IPA with the nuclear factor of activated T-cells (NFAT) pathway which influences cardiac hypertrophy (z-score = 2.24, Table 2). Calmodulin—dependent protein kinase 2, that was found upregulated, is a core mechanism for promoting cardiomyopathy and myocarditis, which are potentially fatal side effects of clozapine [74–76]. Moreover, calcineurin (one of its subunits, PPP3R1, was found increased) directly regulates the activity of CREB and NFAT transcription factors associated with cognitive functions disturbed in schizophrenia [77].

In the CLO12 group, we also found increased abundance of key regulators of protein synthesis: eight ribosomal proteins and translation initiation factor EIF2B4. They were assigned to the eukaryotic initiation factor 2 pathway (z-score = 2.83, Table 2). In olfactory cells derived from schizophrenia patients, English et al. observed a significant reduction in global translation rate together with dysregulation of EIF2, EIF4, and mTOR signaling pathways [78]. However, the same research team detected an increase in the level of translation-related proteins in patients' progenitor cells [79], suggesting a cell type-specific effect. Recent studies have highlighted that an imbalance in the protein synthesis process contributes to neurodevelopmental disorders [80–82]. Proteomic studies on cultured striatal neurons exposed to haloperidol also revealed activation of mTORC1-dependent translation [83]. In the mentioned study, the level of several translation-related proteins, including eukaryotic elongation factor 2 (eEF2), was shown to be elevated. To check if changes in translational machinery are also induced by atypical drugs, the level of eEF2 was tested for two representatives of atypical drugs, risperidone and amisulpride. The increase was shown, however, with less stringent statistical criteria (p < 0.1) [83]. In our study, activation of protein synthesis pathway was found as significant for clozapine, however, there were also some ribosomal proteins upregulated in the haloperidol group (see Supplementary file 2).

Differential proteins from each experimental group were combined in functional networks through the use of IPA. Detailed results are included in Supplementary file 3. Figure 2c shows a network related to neurological diseases that emerged from the CLO12 group. Several proteins included in the network are known to be downregulated in schizophrenia patients, while in our study they were found to be upregulated after clozapine treatment: 14-3-3 protein zeta/delta (YWHAZ), an acidic leucine-rich nuclear phosphoprotein 32 family member A (Anp32a), cytosolic purine 5'-nucleotidase (NT5C2) [84–86]. In addition, the latest study reveals that NT5C2 regulates AMP-activated protein kinase (AMPK) signaling, ribosomal protein S6 (RPS6), and protein translation in neural stem cells [87]. AMPK was also found to be upregulated in the CLO12 group, which confirms the results of Kim et al. [88]. AMPK plays a central role in controlling lipid metabolism and its elevated level may be one of the reasons for metabolic side effects which are induced by clozapine more often than by other antipsychotics [88].

The most prominent finding in the CLO24 experimental group (cells incubated for 24 h with clozapine) was an inhibition of the actin cytoskeleton signaling and Rho proteins signaling (z-score = − 2.00, Table 2), which is consistent with the results of our previous studies conducted on the cortex of rats treated with clozapine [31] and with the outcome of the experiment performed on MK-801 cells by Martins-de-Souza et al. [89]. Proteomic study on oligodendrocytes showed that clozapine and haloperidol affected proteins involved with the actin cytoskeleton and EIF2 signaling [90]. The polymerization of actin is responsible for the synapse plasticity and morphological changes of dendritic spines in response to stimuli [91]. In schizophrenia, synaptic reorganization disturbances are observed in long-term potentiation (LTP) and long-term depression (LTD) [92, 93]. In patients with schizophrenia, altered level of actin-related proteins was frequently reported in the literature, however, the direction of these changes was ambiguous [94–99]. The long-term action of clozapine seems to result in changes of cytoskeletal organisation, probably leading to the restoration of synapse plasticity altered in schizophrenia.

### Suggested Contribution of the mTOR Signaling in the Clozapine Action

**Fig. 3:**
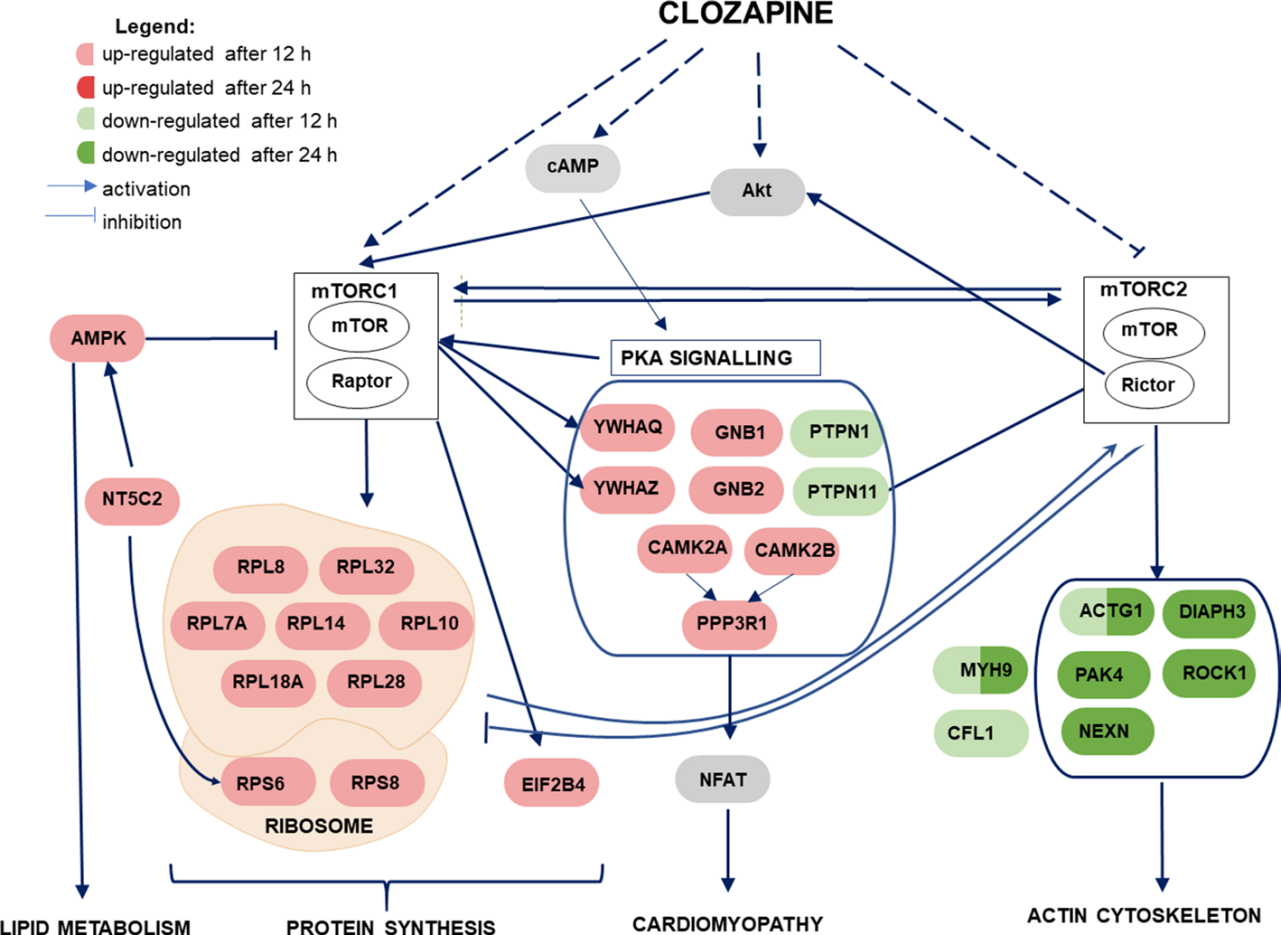
A potential network of interactions of differential proteins identified in PC12 cells under the influence of 12 and 24 h incubation with clozapine. An arrow (↑) indicates stimulation whereas a hammerhead (⊤) indicates inhibition. Some critical pathways have been omitted for clarity

In Fig. 3, the key alterations revealed after clozapine treatment in two time points were integrated into the network on the basis of the literature knowledge. During proteomic dataset analysis, several premises emerged indicating that Akt kinase and mTOR complexes play a superior role in clozapine signal transduction. Recently, disturbances in mTOR signaling are emerging as important factors in schizophrenia and other neurological diseases [100–104].

The mTORC1 controls translation, cell growth, and proliferation, thus increased levels of ribosomal proteins and the eIF-2B translation initiation factor may result from mTORC1 activation [102]. This is reverse to the changes observed in schizophrenic brains. The smaller soma size of patients’ neurons was linked to decreased mTORC1 signaling, while *Akt1* gene expression was downregulated [104]. In mice that display manic-like behaviors, transcriptomic analysis showed downregulation of mTORC1 signaling [105].

Interestingly, the ingenuity upstream analysis tool showed that the upregulation of ribosomal proteins after 12 h incubation with clozapine, may be caused by a decrease in the activity of the Rictor protein (z-score = − 2.13), as it was shown in *Rictor*-knockout mice [106]. Rictor is a key regulatory subunit of mTOR complex 2 (mTORC2) [107]. This complex regulates cellular metabolism as well as stimulates the formation of actin filaments through interaction with RhoA proteins, Rac1, Cdc42 and Cα protein kinase (PKCα) [108]. Therefore, the decrease in the level of actin-binding proteins and in general the promotion of actin depolymerization may be triggered by inhibition of the mTORC2 pathway [109]. Siuta et al. showed that neuronal mTORC2 dysfunction is sufficient to generate cortical hypodopaminergia (postulated as present in schizophrenia) and schizophrenia-linked behaviors (sensorimotor gating deficits) [107]. However, Carson et al. demonstrated that mice with conditional knockout of *Rictor* had reduced anxiety-like behavior, but the negative effect of this knockout was brain development disorder and smaller volume of brain structures [110]. Thus, inhibition of Rictor-controlled pathways induced by clozapine may adjust the dopaminergic balance in the schizophrenic brain. In the later stages, mTORC1 regulates autophagy, mitochondrial function and lipid synthesis [111]. Therefore, dysregulation of mTOR signaling is associated with various diseases such as obesity and type 2 diabetes, which are often cumbersome side-effects of clozapine [112, 113]. Intriguing, the mTOR itself has been found downregulated in the risperidone and haloperidol treated group (see Table 2). Moreover, regulation of translation-related machinery as well as actin cytoskeleton signaling was not recognized for these drugs as significant by IPA. This looks contrary to the study of Bowling et al. showing the activation of mTORC1-dependent translation after treatment with haloperidol in cultured striatal neurons [83]. However, the other group found that olanzapine (atypical antipsychotic drug), but not haloperidol activates mTORC1 signaling in rat primary hippocampal neurons [114]. Similarly, Deslauriers et al. reported no significant effect on mTOR activation for haloperidol and amisulpride in SH-SY5Y human neuroblastoma cells [115]. Thus, it seems that there is no universal mechanism regarding impact of haloperidol on mTOR signaling. This may depend on the model used for the study as well as experimental conditions, such as drug concentration or duration of treatment.

### Limitations/Rationale of Using PC12 Cells as a Model Research System

To study antipsychotic drug action, we used the PC12 cell line, which is one of the most commonly used in neuroscience research. The main limitation of the in vitro cell culture system is the fact that it definitely cannot reflect interactions between different cell types and between cells and extracellular matrix which take place in the brain. There is no feedback from other types of cells and body fluids. However, we chose PC12 cell line as the simplest model to eliminate the complexity of the nervous system in the study. The advantages of in vitro cell culture systems are controlled environment and reduced experimental variation which could reliably yield better quality, more accurate results. In our previous experiments, we studied antipsychotic drug action on rat brain tissue and the proteome changes revealed were very subtle [31, 116]. Here, we reached for naïve PC12 cell line, to work with homogenous cell population to increase the chances of revealing the potential changes in protein profile. However, detected changes were still small, what unequivocally proves that antipsychotic drugs’ impact on the protein profiles is very subtle. We also did not treat PC12 cells with nerve growth factor to avoid additional variables. The similar model was used by Tejedor-Real et al. They observed similar changes in dopamine synthesis pathway (tyrosine hydroxylase) in the rat brain and PC12 cells after clozapine administration [21]. PC12 was also used as dopamine cell model [117]. PC12 cells express a high level of glucocorticoid receptors therefore they were utilized for hyperactivation of hypothalamic–pituitary–adrenal (HPA) axis relevant studies [118].

## Concluding Remarks

Understanding the molecular mechanism of antipsychotic drugs remains an urgent issue that has been studied for the last 60 years without conclusive outcomes. However, the development of analytical techniques brings us closer to this goal. In our study, we applied two high-throughput proteomic approaches which enabled to explore the antipsychotic impact on cells at the pathway level. As alterations in protein profile revealed in our study have often not been linked to antipsychotics before, we also discuss it in the context of schizophrenia. All tested drugs seemed to stimulate a beneficial process of reducing inflammation, however, by activating different biochemical pathways. Additionally, for risperidone, pathway analysis showed inhibitory effect of acute phase response. In the case of haloperidol, the changes in the proteome indicated inhibition of ciliary trophic factor signaling. The 12-h incubation with clozapine caused up-regulation of protein kinase A signaling and translation machinery. After 24 h of treatment with clozapine, the inhibition of the actin cytoskeleton signaling and Rho proteins signaling was revealed. The observed changes suggest a central role of kinase mTOR in clozapine signal transduction. Decreased abundance of actin-binding proteins could be a result of inhibition of mTORC2 pathway, while stimulation of translation could be an effect of the mTORC1 pathway activation. On the other hand, the upregulation of calmodulin-dependent protein kinase 2 and AMPK signaling could be linked with adverse effects of clozapine administration. The most interesting question during the investigation was to catch the unique effectiveness of clozapine and the study revealed the importance of mTOR signaling for this drug. However, further biochemical analyses are required to explore this issue.

## Supplementary Information

Below is the link to the electronic supplementary material.

Supplementary file1 (DOCX 229 kb)

Supplementary file2 (XLSX 106 kb)

Supplementary file3 (XLSX 417 kb)

## Data Availability

The MS proteomics data have been deposited to the ProteomeXchange Consortium via the PRIDE [1] partner repository with the dataset identifier PXD014422.

## Abbreviations

AGC: Automatic gain control
Akt: Protein kinase B
AMP: Adenosine monophosphate
AMPK: AMP-activated protein kinase
CNTF: Ciliary neurotrophic factor
IL1RN: Interleukin-1 receptor antagonist protein
IPA: Ingenuity Pathway Analysis
IS: Internal standard
MAP2K2: MAP kinase kinase 2
mTOR: Mammalian target of rapamycin
mTORC1: MTOR complex 1
mTORC2: MTOR complex 2
NCE: Normalized collision energy
NFAT: Nuclear factor of activated T-cells
NT5C2: Cytosolic purine 5′-nucleotidase
MMP14: Matrix metalloproteinase-14
PIK3C3: PI3-kinase type 3
PKA: Protein kinase A
PTPN11: Tyrosine-protein phosphatase non-receptor type 11
SASP: Senescence-associated secretory phenotype
SERPINE1: Plasminogen activator inhibitor 1
SHC1: SHC-transforming protein 1
TGF-β1: Transforming growth factor beta 1
TNF: Tumor necrosis factor
