# Supplementary material for: Proteome Analysis of PC12 Cells Reveals Alterations in Translation Regulation and Actin Signaling Induced by Clozapine
Source: Neurochem Res. 2021 May 23;46(8):2097–111. doi: 10.1007/s11064-021-03348-4 (PMC8254727; doi:10.1007/s11064-021-03348-4)
Supplement: Supplementary file 1 — Supplementary file1 (DOCX 229 kb) [file 11064_2021_3348_MOESM1_ESM.docx]

**Proteome analysis of PC12 cells reveals alterations in translation regulation and actin signaling induced by clozapine**

*Neurochemical Research*

Urszula Jankowska^1^, Bozena Skupien-Rabian^1^, Bianka Swiderska^2^, Gabriela Prus^3^, Marta Dziedzicka-Wasylewska^3^_,_ Sylwia Kedracka-Krok^3^

*^1^ Malopolska Centre of Biotechnology, Jagiellonian University, Krakow, Poland*

*^2^ Mass Spectrometry Laboratory, Institute of Biochemistry and Biophysics Polish Academy*

*of Sciences, Warsaw, Poland*

*^3^ Department of Physical Biochemistry, Faculty of Biochemistry, Biophysics and Biotechnology, Jagiellonian University, Krakow, Poland*

Correspondence: urszula.jankowska@uj.edu.pl

**SUPPLEMENTARY MATERIALS AND METHODS**

**1.1 iFASP procedure**

Protein digestion followed by peptide labelling was performed according to the iFASP procedure (isobaric mass tagging with filter-aided sample preparation) [1] with some modifications. The protein mixture (50 μg) was brought to 200 µl with an urea solution containing 8 M urea in 50 mM ammonium bicarbonate (ABC) and reduced with dithiothreitol (DTT) at a final concentration of 50 mM at room temperature for 20 min. The samples were transfer to spin columns with a 30-kDa membrane cutoff (Vivacon500, Sartorius Stedim, Germany) and washed twice with the urea solution. Alkylation was carried out with 55 mM iodoacetamide (IAA) in urea solution for 20 min in the dark. Three washes with urea solution and three washes with 100 mM triethylammonium bicarbonate (TEAB) were performed. Trypsin solution (Promega) was added at an enzyme to protein ratio of 1:50. The columns were placed in a wet chamber to prevent evaporation during overnight incubation at 37 °C. Peptides were labelled on spin columns using 8-plex iTRAQ reagent (Applied Biosystems). Each iTRAQ reagent was diluted in 120 μl isopropanol, then divided into two parts and added to two different samples according to the labelling scheme shown in Table S1 below. The mixture was incubated at room temperature for 2 h with shaking at 600 rpm. Peptides were collected through two washes with 50 mM ABC and one wash with 0.5 M NaCl. The collected filtrates were combined according to Suppl. Table S1. Salts and excess reagents were removed by solid phase extraction on C18 cartridges (Empore Extraction Disk Cartridges, bed I.D. 7 mm, volume 3 ml, Sigma).

**1.2 Fractionation of peptides by IEF**

To reduce sample complexity before MS analysis, peptides were fractionated according to p*I*. The iTRAQ-labelled peptides were dissolved in 450 μl of rehydration buffer (10% glycerol, 1% 3-10 L IPG buffer, 0.001% bromophenol blue) and loaded into 24 cm linear pH 3-10 Immobiline DryStrips (GE Healthcare). After overnight passive rehydration, the strips were wrapped with parafilm and placed in a PROTEAN® i12™ IEF System (Bio-Rad). IEF was performed with a current limit of 50 µA according to following running conditions: 100 V for 10 min, gradient to 2,000 V for 10 h and 2,000 V for up to 60,000 Vh. The strips after IEF were frozen at -80°C, cut into 34 fragments and transferred to separate tubes. Peptides were sequentially extracted from the gels with: 1) 0.1% TFA, 50 mM ABC, 2) 0.1% TFA, 50% ACN, 50 mM ABC, 3) 100% ACN. The peptides were vacuum-dried and purified on C18 StageTips [2].

[1] G. S. McDowell, A. Gaun, H. Steen, *J. Proteome Res.* **2013**, *12* (8), 3809–12. DOI: 10.1021/pr400032m.

[2] J. Rappsilber, M. Mann, Y. Ishihama, *Nat. Protoc.* **2007**, *2* (8), 1896–906. DOI: 10.1038/nprot.2007.261.

**SUPPLEMENTARY FIGURES**

Abbreviations used in figures and tables:

CLO12: clozapine 12 h

CLO24: clozapine 24 h

RIS12: risperidone 12 h

RIS24: risperidone 24 h

HAL12: haloperidol 12 h

HAL24: haloperidol 24 h

**Table S1. Scheme of labelling samples with 8-plex iTRAQ.** Two passages of PC12 cells were combined prior to labelling: (a) passage 1 and 3; (b) passage 2 and 5; (c) passage 4 and 6. Three samples from each experimental group (marked with a coloured background) were subjected to a quantitative analysis in the Scaffold program. Table contain number of identified peptides and proteins from each IEF strip.

| **iTRAQ tag** | **IEF Strip 1** | **IEF Strip 2** | **IEF Strip 3** | **IEF Strip 4** |
| --- | --- | --- | --- | --- |
| **113** | internal standard | internal standard | internal standard | internal standard |
| **114** | CON12a | HAL24a | CON24b | RIS12c |
| **115** | CLO12a | CON12b | CLO24b | HAL12c |
| **116** | RIS12a | CLO12b | RIS24b | CON24c |
| **117** | HAL12a | RIS12b | HAL24b | CLO24c |
| **118** | CON24a | HAL12b | CON12c | RIS24c |
| **119** | CLO24a | CON24b | CON24b | CLO24c |
| **121** | RIS24a | CLO24b | CLO12c | HAL24c |
| # iTRAQtag peptides (FDR < 1%) | 37 720 | 36 326 | 22 596 | 22 986 |
| #identified proteins (clusterd) | 5 341 | 5 186 | 3 630 | 3 936 |

**Table S2. Scheme of labelling samples with cyanine dyes prior to DIGE.**

| **DIGE GEL** | **Cy2** | **Cy3** | **Cy5** |
| --- | --- | --- | --- |
| **1** | internal standard | CON12 | CLO12 |
| **2** | internal standard | RIS12 | HAL12 |
| **3** | internal standard | CON24 | CLO24 |
| **4** | internal standard | RIS24 | HAL24 |
| **5** | internal standard | CLO12 | CON12 |
| **6** | internal standard | HAL12 | RIS12 |
| **7** | internal standard | CLO24 | CON24 |
| **8** | internal standard | HAL24 | RIS24 |
| **9** | internal standard | CON12 | CLO12 |
| **10** | internal standard | RIS12 | HAL12 |
| **11** | internal standard | CON24 | CLO24 |
| **12** | internal standard | RIS24 | HAL24 |
| **13** | internal standard | CLO12 | CON12 |
| **14** | internal standard | HAL12 | RIS12 |
| **15** | internal standard | CLO24 | CON24 |
| **16** | internal standard | HAL24 | RIS24 |
| **17** | internal standard | CON12 | CLO12 |
| **18** | internal standard | RIS12 | HAL12 |
| **19** | internal standard | CON24 | CLO24 |
| **20** | internal standard | RIS24 | HAL24 |
| **21** | internal standard | CLO12 | CON12 |
| **22** | internal standard | HAL12 | RIS12 |
| **23** | internal standard | CLO24 | CON24 |
| **24** | internal standard | HAL24 | RIS24 |


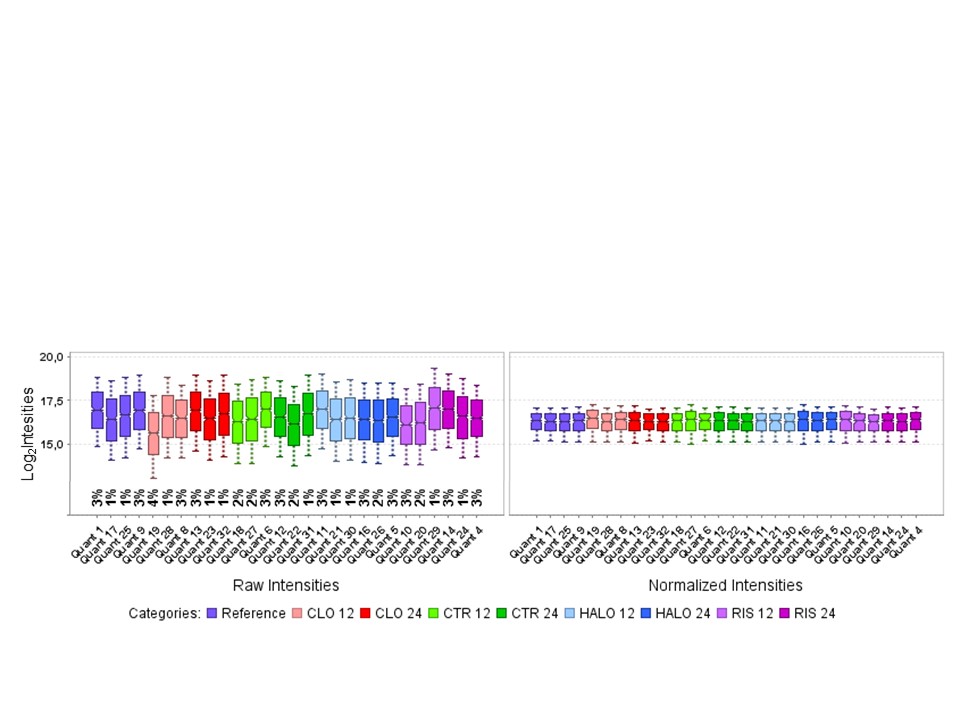


**Figure S1.** Raw and normalized peptide intensities in iTRAQ experiment; whiskers illustrate 5th-95th percentiles. Datasets were initially normalized using a peptide mean intensity based normalization in Scaffold software.

**
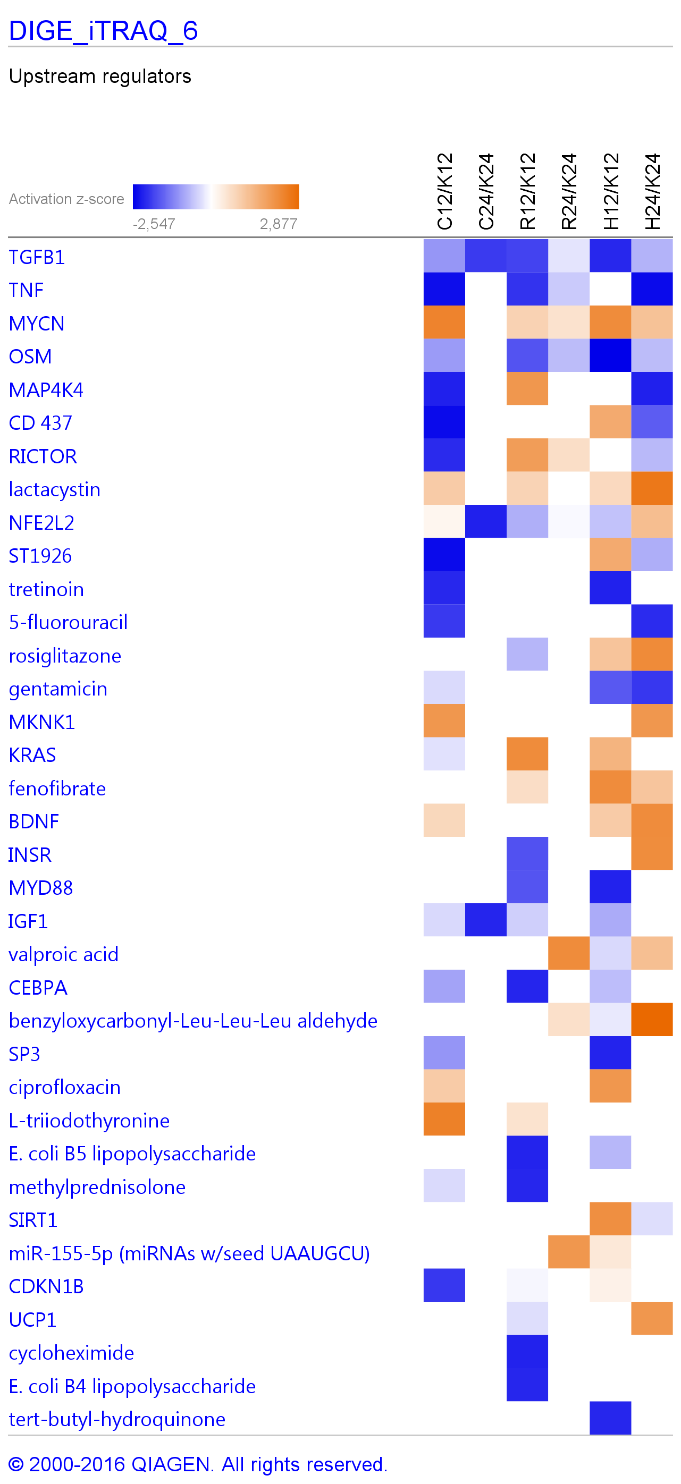
**

**Figure S2.** Upstream regulators of the proteome changes induced by incubation with clozapine, risperidone and haloperidol for 12 and 24 h. Based on alterations of differential proteins, the activity of a given regulator was predicted to be inhibited (blue) or increased (orange) by IPA software.
